# Child development in the context of biological and psychosocial hazards among poor families in Bangladesh

**DOI:** 10.1371/journal.pone.0215304

**Published:** 2019-05-06

**Authors:** Sarah K. G. Jensen, Fahmida Tofail, Rashidul Haque, William A. Petri, Charles A. Nelson

**Affiliations:** 1 Department of Pediatrics, Boston Children’s Hospital, Boston, Massachusetts, United States of America; 2 Department of Pediatrics, Harvard Medical School, Boston, Massachusetts, United States of America; 3 Nutrition and Clinical Services Division, ICDDR,B, Dhaka, Bangladesh; 4 Infectious Diseases Division, ICDDR,B, Dhaka, Bangladesh; 5 Infectious Diseases & International Health, University of Virginia, Charlottesville, Virginia, United States of America; 6 Harvard Graduate School of Education, Harvard College, Cambridge, Massachusetts, United States of America; University of North Carolina at Chapel Hill, UNITED STATES

## Abstract

It is well established that low resource environments early in life can predispose children to adverse health and compromised developmental outcomes. We explore possible mechanistic pathways underlying poor developmental outcomes in children growing up in a low resource setting in urban Bangladesh. We tested associations between psychosocial risks, namely maternal distress and poor caregiving experiences, and biological risks, namely poor growth (HAZ) and inflammation (C-reactive protein: CRP), and children’s developmental outcomes. Child development was measured using the Mullen Scales of Early Learning (MSEL) at 6 and 27 months in one cohort, and using the MSEL and Wechsler Preschool and Primary Scale of Intelligence (WPPSI) at 36 and 60 months respectively in another cohort. In the younger cohort, we found that more inflammation (estimated by the child’s CRP level at four months) predicted lower receptive language scores at 6 months, while more frequent caregiving interactions predicted higher receptive language scores at 6 months. In the older cohort, we found that at 27 months, a child’s growth measured by his or her current HAZ was positively associated with gross motor, visual reception, receptive language, and expressive language scores. In the oldest cohort, we found that higher HAZ and more frequent stimulating activities in the home predicted higher motor and language scores, whereas more inflammation (as estimated by CRP over the first two years of life) predicted lower motor scores at 36 months. At 60 months, we found that HAZ and caregiving experiences were positively associated with verbal IQ, whereas inflammation was negatively associated with verbal IQ. This work identifies malnutrition, inflammation, and caregiving as potential sites of intervention to improve neurodevelopment in children growing up in global poverty.

## Introduction

Globally, the scope of childhood poverty is troubling with nearly 20% of children in the developing world living in extreme poverty, defined by the World Bank as living on less than $1.90 per day [1]. Poverty is a well-established risk factor for poor developmental outcomes, including delayed achievement of developmental milestones, poor performance on cognitive tests resulting in poor school performance, and poor physical health. Reducing the adverse consequences of childhood poverty is a global health priority, and research to increase our understanding of the specific mechanisms underlying the association between poverty and child outcomes is an important step towards the development of effective interventions. While the commonly used definitions of poverty use financial metrics, these metrics can only approximate the actual mechanistic pathways that underlie adverse childhood development, including complex environmental and psychosocial exposures that have a more direct impact on children. Examples of poverty-related exposure that may directly impact child development include biological risks such as inadequate nutrition and childhood infections that precipitate inflammation and impact nutrition, as well as psychosocial risks, such as family and caregiver stress and parental mental health problems, which can impact both the amount and quality of cognitive stimulation to which a child is exposed. Previous studies have characterized the effects of biological and psychosocial risk exposures on various aspects of child development, yet most studies examine single risk exposures in isolation without taking the correlated and cumulative nature of risk exposures into account. With regard to biological risks, poor childhood growth (often measured as low standardized height-for-age: HAZ) has been consistently shown to predict poorer cognitive, motor, and language outcomes in children [2, 3]. In low resource settings, low HAZ tends to be the result of malnutrition and infectious disease, two common risk exposures with possible impacts on neural development [2]. Malnutrition may be caused by inability to purchase or access diverse and nutritious food, and deficiency of macro and micronutrients can have profound impacts on neural development and functioning [2]. Infectious diseases, including enteric infections such as diarrheal illness, are common in low-resource settings where there is limited access to water sanitization equipment and public sewage systems, and enteric infections have been shown to predict poorer developmental outcomes in infants and school-aged children [4]. Evidence from low income countries suggests that adverse effects of enteric infections on children’s cognitive outcomes are driven by inflammatory processes, indexed by an increased production of inflammatory cytokines and C-reactive protein (CRP) [5,6]. Inflammation may affect child development through different pathways including direct effects of inflammation on neural growth and functioning, sickness behaviours in the child that may delay learning, and interactions with other biological processes in the body. Enteric infections may, for instance, compound baseline malnutrition to impact growth [7]. With regard to psychosocial risks, previous research has highlighted the impact of stress, parental mental health illness, and the social caregiving environment as a key mechanism through which poverty impacts early child development [8, 9, 10, 11, 12]. Studies investigating maternal depression have revealed its impact on childhood development as early as three months [13] and throughout childhood [14].

**Fig 1:**
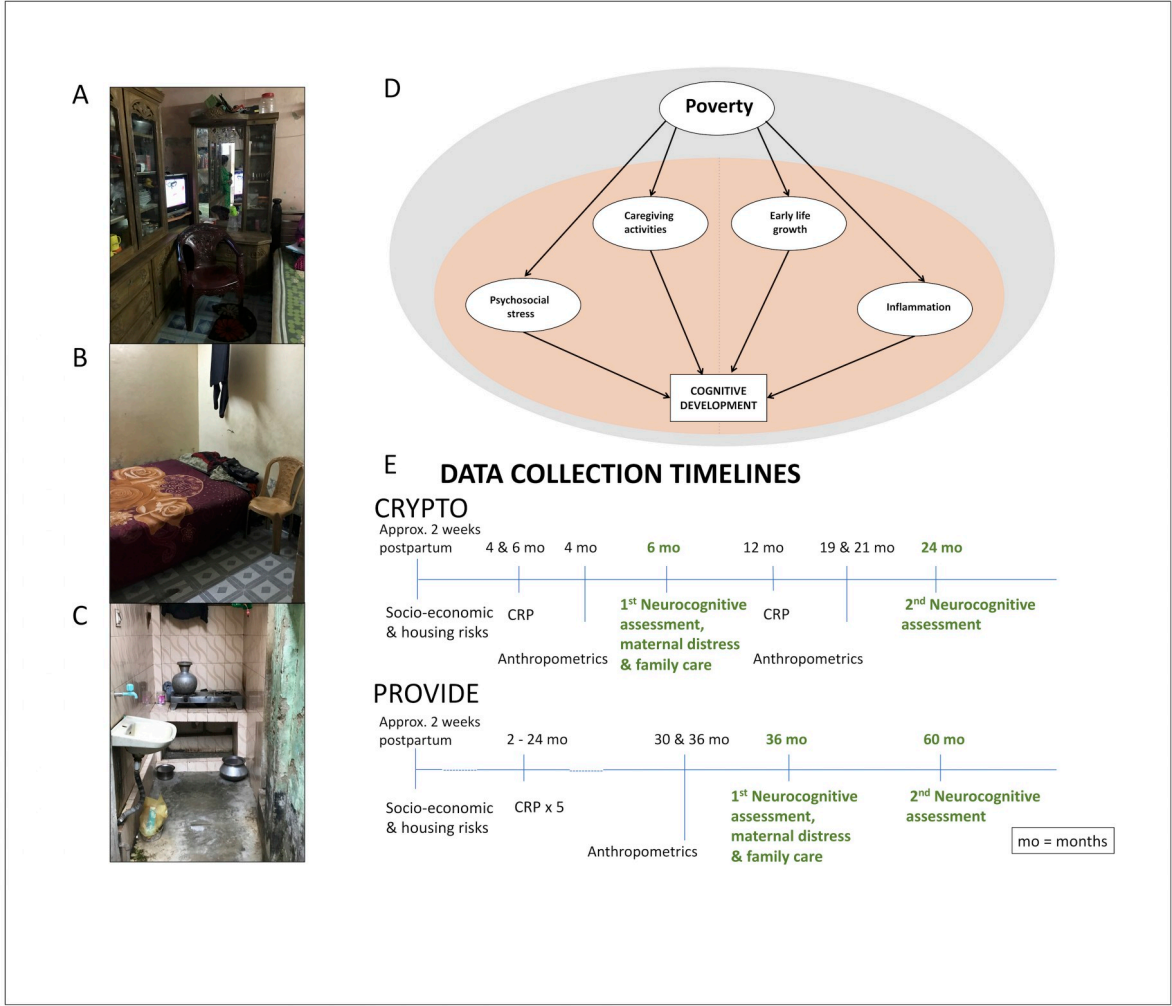
Study context, hypotheses and design. (A-C) Images taken during home visits in the Mirpur community in Dhaka, Bangladesh showing a standard one-room family home, ad standard shared cooking facilities. (D) The theoretical model highlighting maternal distress, family caregiving activities, early growth, and inflammation as important mediators of the effects of poverty on early child development. (E) Timeline for data collection.

Given the complex interplay of risk exposures that tend to co-occur in low-income and low resource homes, and the consistent evidence identifying these exposures as potential threats to child development, increased knowledge about the independent contribution of individual biological and psychosocial risks to poor child development is key to the development of successful interventions to support healthy child development. This study aims to examine developmental associations between specific risk factors that tend to co-occur with poverty and that can be targeted by specific interventions to support children who grow up in high-poverty settings. We use data from two cohorts of children from Dhaka, Bangladesh to examine four risk exposures that we hypothesize may affect child development in this low resource setting, namely child growth (used as an indicator of the child’s nutritional environment and health and measured using children’s HAZ), inflammation (measured using serum C-reactive protein (CRP) levels in blood), maternal distress (measured using maternal reports of stress and depressive symptoms), and the degree of stimulation a child receives at home, measured as parent reported family caregiving activities (See Fig 1). Cognitive, motor, and language outcomes were assessed when children were 6 and 27 months old in one cohort, and 36 and 60 months old in the other cohort.

## Methods

### Study design

We used data from two cohorts of children who took part in the Bangladesh Early Adversity Neuroimaging (BEAN) study, a collaboration between Boston Children’s Hospital, the University of Virginia, and the International Centre for Diarrhoeal Disease Research, Bangladesh (ICDDR,B). Data were collected between 2015 and 2018. Children were recruited from a medical clinic in the Mirpur district of Dhaka, Bangladesh. Exclusion criteria limited the sample to infants and children born > = 34 weeks gestation, with no history of neurological abnormalities or traumatic brain injury, no known genetic disorders, and no known visual or auditory delays or impairments. Children from the “Cryptosporidium Burden Study” (CRYPTO) underwent behavioral assessments at 6 months (n = 130) and 27 months (n = 104). Children from the “The Performance of Rotavirus and Oral Polio Vaccines in Developing Countries” (PROVIDE) cohort underwent developmental and cognitive assessments at 36 months (n = 130) and 60 months (n = 122). Descriptive information about the samples is provided in Table 1. Ethical approval was obtained from research review and ethics review committees at The International Centre for Diarrhoeal Disease Research in Bangladesh and the Institutional Review Boards at Boston Children’s Hospital and the University of Virginia. All parents of children enrolled in the study gave written consent for themselves and their child to participate in the study.

**Table 1:** Descriptive information about the two cohorts.

	CRYPTO		PROVIDE		T-test
Socioeconomic characteristics	Number	%	Number	%	
Monthly household income [US$]	15149 (SD = 9612) [$185]		12774 (SD = 8981) [$155]		t(258) = 2.06,p = 0.041
Income pr. household member pr. day [US$]	100 (SD = 46.38) [$1.23]		84 (SD = 64.72) [$1.02]		t(258) = 2.22,p = 0.027
Families with < 1.90 US$ per. household member pr. day	116	89%	120	92%	
Average paternal years of education	4.5 (SD = 3.80)		4.7 (SD = 3.83)		t(258) = -0.60,p = 0.547
Number of fathers with no education	41	32%	36	31%	
Average maternal years of education	4.5 (SD = 3.42)		4.2 (SD = 3.61)		t(258) = -0.67,p = 0.504
Number of mothers with no education	32	25%	41	34%	
**Housing risks**					
Average number of housing risks [0–9]	3.45 (SD = 1.41)		3.88 (SD = 1.54)		t(258) = -2.30,p = 0.022
*Flooring [Earth (vs*. *floor is cement*, *bamboo or wood)]*	*1*	*1%*	*12*	*10%*	
*Walls [Tin*, *bamboo or straw (vs*. *cement or brick)]*	*14*	*11%*	*36*	*29%*	
*Roof [Tin or straw (vs*. *finished roof) ]*	*83*	*64%*	*102*	*82%*	
*Cooking gas [Family has gas (vs*. *no gas)]*	*46*	*36%*	*40*	*32%*	
*Toilet (type) [Latrine or more primitive (vs*. *septic tank)]*	*87*	*67%*	*46*	*37%*	
*Shared toilet (vs*. *private)*	*94*	*73%*	*112*	*90%*	
*Open drain in front of home (vs*. *none)*	*43*	*33%*	*54*	*43%*	
*Water source [Non-municipality supply]*	*5*	*4%*	*3*	*2%*	
*Crowding (> 3 people pr*. *room)*	*70*	*54%*	*80*	*64%*	
**Absence of assets**					
Average number of assets [0–13]	5.05 (SD = 1.62)		5.46 (SD = 2.00)		t(258) = -1.80,p = 0.073
*Number of families with no phone*	*4*	*3%*	*22*	*18%*	
*Number of families with no almeria*	*42*	*32%*	*78*	*63%*	
*Number of families with no table*	*104*	*81%*	*83*	*66%*	
*Number of families with no chair*	*80*	*62%*	*70*	*57%*	
*Number of families with no bench*	*120*	*93%*	*117*	*94%*	
*Number of families with no clock*	*29*	*23%*	*44*	*36%*	
*Number of families with no bed*	*7*	*5%*	*2*	*2%*	
*Number of families with no radio*	*128*	*99%*	*122*	*98%*	
*Number of families with no television*	*22*	*17%*	*27*	*22%*	
*Number of families with no bicycle*	*125*	*97%*	*121*	*98%*	
*Number of families with no motor cycle*	*129*	*100%*	*122*	*98%*	
*Number of families with no sewing machine*	*115*	*89%*	*117*	*94%*	
*Number of families with no fan*	*4*	*3%*	*3*	*2%*	
**Food insecurity**					
Degree of food security [1–4, see below]	3.52 (SD = 0.80)		2.81 [SD = 0.81]		t(258) = 7.27,p<0.001
*Report whole year deficit of good*	*5*	*4%*	*4*	*3%*	
*Report occasional deficit of food*	*8*	*6%*	*44*	*36%*	
*Report neither deficit nor surplus of food*	*31*	*24%*	*51*	*41%*	
*Report surplus of food*	*85*	*66%*	*25*	*20%*	
**Maternal distress**					
Maternal stress, first visit (6 or 36 m)	15.85 (SD = 8.81)		14.50 (SD = 8.10)		t(257) = 1.28,p = 0.202
Maternal stress, second (27 or 60 m)	12.37 (SD = 7.94)		12.43 (SD = 7.93)		t(230) = -0.050,p = 0.960
Maternal depressive symptoms, first visit (6 or 36 m)	7.05 (SD = 5.22)		6.83 (SD = 4.32)		t(257) = 0.362,p = 0.717
Maternal depressive symptoms, second visit (27 or 60 m)	3.41 (SD = 4.38)		3.18 (SD = 3.92)		t(230) = 0.427,p = 0.670
**Stimulating caregiving activities in the home**					
Family care [number activities] first visit (6 or 36 m)	4.07 (SD = 1.83)		8.08 (SD = 2.78)		
Family care [number activities] second visit (27 or 60 m)	5.68 (SD = 1.85)		5.68 (SD = 1.85)		t(230) = -0.137,p = 0.891
**Child characteristics**					
HAZ at first visit (6 or 36 m)	-1.06 (SD = 0.91)		-1.42 (SD = 0.86)		
Number of stunted children at first visit	21	0.16	42	0.34	
CRP level in blood at 6 m CRYPTO, 24 m PROVIDE (mg/L)	3.90 (SD = 6.02)		6.21 (SD = 12.74)		
No. of times the child had an elevated CRP first two years [range, 0–5]*	-	-	2.51 (SD = 1.30)		

m = months; SD = Standard deviation; EPDS = Edinburg Postnatal Depression Scale; HAZ = standardized height-for-age; CRP = C-reactive protein.

*Elevated CRP was defined as a child exceeding the 50^th^ percentile within the sample.

### Variables of interest

An overview of the variables included in this study and the timeline for the data collection is included in Fig 1. Measures of socio-economic status (income, education), maternal distress (depression and stress), and family care (number of stimulating activities) were assessed via interviews with study parents conducted by local, native staff who were train on the measures and interview techniques before the study started.

#### Poverty

Household poverty was assessed via information obtained from parental interviews and home visits during which housing maternal including building materials and sanitation and the presence of various assets was assessed and noted by an observer. These interviews and home visits were conducted shortly after the child was born. Household poverty was defined as a latent factor with three indicators. The first indicator was income-to-needs quartiles calculated as the monthly household income divided by the number of people in the household. The second indicator was a cumulative index of the family’s housing risks as observed in their homes, and the third indicator was an index of family assets observed in the home or reported by parents. Information about household income and prevalence rates for housing risks and assets are provided in Table 1. The three indices were highly correlated with each other and loaded on a common factor score referred to as “Poverty” (see supplemental information for more details of the confirmatory factor analysis).

#### Maternal distress

Maternal distress was assessed using standard questionnaires administered in oral interviews with mothers at the time of the child’s developmental assessments (6 months and 27 months in CRYPTO; 36 and 60 months in PROVIDE). Interviews were conducted in Bangla by local, trained interviewers to overcome potential confounding by maternal literacy. Questions were field tested with mothers from the community before data collection started and interviewers underwent rigorous training before interviewing study mothers. Maternal distress was measured as a latent factor score using summary scores from two maternal questionnaires, namely a Bangla version of the Edinburg Postnatal Depression Scale [15] and the Perceived Stress Scale [16]. These scales were highly correlated and loaded well on a common factor that we refer to as “maternal distress” (see supplemental information for more details of the confirmatory factor analysis).

#### Family care

Family care was assessed during interviews with mothers at the time of the developmental assessments using questions from the Family Care Indicators (FCI) from UNICEF’s Multiple Indictor Cluster Survey [17] and additional questions to assess stimulating activities that the mother, father, or another “caregiver” engaged in with the child within the last 30 days). Activities included, for instance, play, singing, and reading activities. The FCI has been widely used to assess the quality of children’s home environments in low and middle-income countries, including Bangladesh [18].

#### HAZ

Children’s height was measured in centimetres and converted into an age referenced z-score called the HAZ using the World Health Organization’s Anthro Plus software (version 3.2.2). We calculated average HAZ scores over two time points to minimize measurement error. In the analyses involving the CRYPTO cohort, we used an average of the HAZ scores collected when the child was 4 and 6 months in our analysis of outcomes at 6 months and an average of the HAZ scores collected at 19 and 21 months in our analysis of outcomes at 27 months. For all analyses involving PROVIDE, we averaged HAZ scores collected at 30 and 36 months.

#### Inflammation

Inflammation was measured as the concentration of C-reactive protein (CRP) in peripheral blood in mg/L obtained using an enzyme linked immunosorbent assay (ELISA) (Immunidiagnostik AG). CRP is a protein that is synthesized in the liver in response to inflammation. Children from CRYPTO had blood drawn to assess CRP levels at 4 months and 12 months. Because of a negative skew, we used a log transformation to create a normally distributed continuous variable in line with previous publications [5]. We used the log transformed CRP concentration assessed at 4 months as a predictor of cognition at 6 months, and the log transformed CRP concentration at 12 months as a predictor of cognition at 27 months. Children from PROVIDE had five CRP assessments obtained between the ages of 2 and 24 months. This allowed us to create a cumulative estimate of children’s “persistent inflammatory burden.” In line with previous publications from this cohort, we created a cumulative score by summing up the number of times the child’s CRP level exceeded the 50th percentile within the sample [19]. We note the due to the differences in the frequency at which CRP was assessed the variable used in the CRYPTO cohort likely reflects acute inflammation 2–12 months prior to the cognitive assessment, whereas the CRP variable in PROVIDE reflects persistent or chronic inflammation.

#### Child development

**Fig 2:**
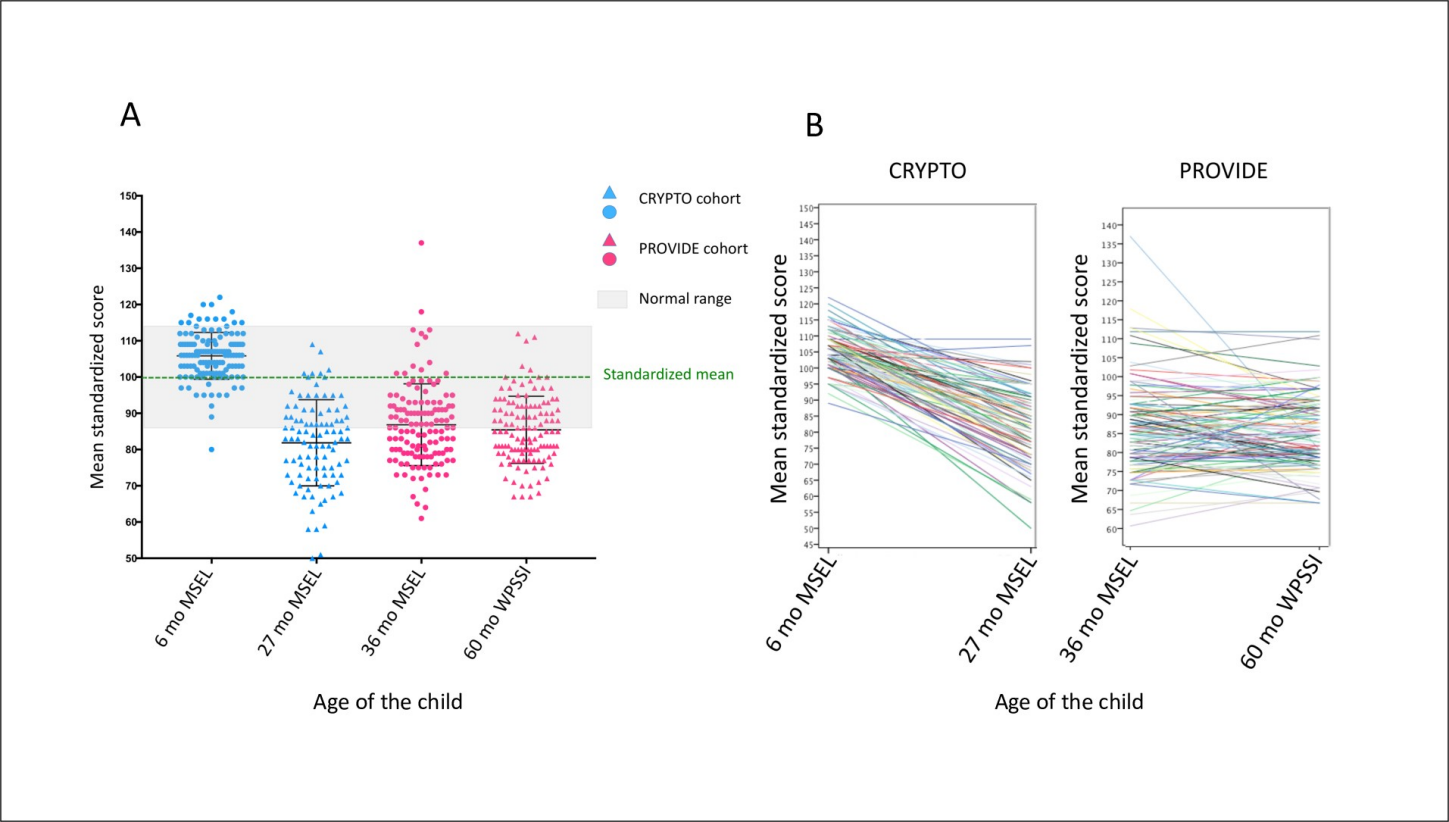
Child developmental outcomes. **(**A) Overview of children’s standardized performance. (B) Spaghetti plots of changes in children’s performance across the two time points in each cohort. Note change in the developmental outcome measure from MSEL at 36 to WPPSI at 60 months.

Child development was assessed using an adapted version of the Mullen Scales of Early Leaning (MSEL) [20] administered when children were 6, 27, and 36 months old. The MSEL assesses child outcomes across five developmental domains: gross motor, fine motor, visual reception, receptive language, and expressive language. The MSEL has previously been used in other low-income countries and local staff adapted the protocol by substituting potentially unfamiliar images and questions with objects and examples that Bangladeshi children would recognize (see online supplement for an overview of adaptations). Because there is no standardization relevant to Bangladeshi children for the MSEL, children’s raw scores were standardized within the sample in each cohort and at each time point to obtain standardized z-scores that were used in the analyses. A composite t-score based on children’s combined motor, language, and visual reception score was used to illustrate children’s performance level in Fig 2 and obtained using the standardized MSEL t-score. At 60 months old, we administered the Wechsler Preschool and Primary Scale of Intelligence (WPPSI) [21] to assess developmental outcomes focusing on two subscales, namely verbal and performance IQ. We changed instrument at 60 months because the MSEL is designed to evaluate skills anticipated to reach a ceiling effect.

#### Covariates

Gestational age (in weeks) was added as an estimate of perinatal risk, which may correlate with and compound both the risk exposures and developmental outcomes.

### Statistical analyses

The theoretical model was tested using structural equation modelling (SEM) with latent and observed variables. SEM has several advantages over other regression techniques, including the ability to a) incorporate both latent and observed variables, b) simultaneously test multiple predictive relationships with multiple outcomes while accounting for covariance between measures collected at the same time-point, c) examine both direct and indirect effects, and d) provide a global measure of how well the full model represents the data. Model fit was evaluated based on a non-significant χ ^2^ (*p*>0.05), the confirmatory fit index (CFI) > 0.95, the root mean square residual (SRMR) < 0.08, and The Root Mean Square Error of Approximation (RMSEA) <0.06 [22]. Individual paths were assessed using a statistical threshold of *p*<0.05. Indirect effects are estimated using the MODEL INDIRECT command in Mplus and were bootstrapped 10,000 times with bias-corrected confidence intervals. All analyses were conducted in Mplus version 7 [23]). Missingness was low (<10%) in both cohorts. Models were estimated using full maximum likelihood estimation with robust errors allowing for inclusion of respondents with missing data on the independent variables. This is preferable because list-wise deletion of cases with partial data can increase sample bias [24].

### Model building strategy

A separate model was specified for each of the four time points (6, 27, 36, and 60 months) at which developmental outcomes were assessed. Each model was tested with and without direct effects of household poverty on children’s developmental outcomes and these nested models were compared using a chi-square difference test. Since neither of the more complex models with direct effects of poverty were found to fit the data better we selected the more parsimonious models without these direct paths. Indirect effects of poverty via the hypothesized mediators were assessed whenever a mediating variable (inflammation, HAZ, family care, or maternal distress) showed a significant relationship with both poverty and a child outcome (*p*<0.05).

## Results

### Descriptive statistics

Descriptive information and comparisons between the two cohorts are provided in Table 1. The majority of the study families lived in extreme poverty defined by the World Bank as living on less than $1.9 per household member per day [1]. The average family had four housing risks and five assets. Images from the homes of study families are shown in Fig 1. Mothers reported moderate levels of stress and depression compared with rates observed in studies conducted in similar settings [15, 16]. Parents, on average, reported having engaged in more activities with their children at the 27- and 36- month time-points. The average HAZ among the infants at 6 months of age was -1.13, and 16% met criteria for stunting (an HAZ <-2.0). The average HAZ among children at 36 months of age was -1.42, and 34% met criteria for stunting. The level of inflammation was high as indicated by mean CRP scores of 3.90 (SD = 6.02) at 6 months in CRYTO and 6.21 (SD = 12.74) at 24 months in PROVIDE.

Children’s mean standardized composite t-scores on the MSEL cognitive composite and full-scale IQ scores are illustrated in Fig 2. In the youngest cohort, we found that children had a mean MSEL composite t-score of 105.85 (SD = 6.46) at 6 months, which dropped to 81.86 (SD = 11.88) at 27 months. This drop was statistically significant: t(96) = 21.53, p<0.001. In the oldest cohort, we found that children had a mean composite t-score of 86.84 (SD = 11.29) at 36 months and a mean full-scale IQ score of 85.44 (SD = 9.25) at 60 months.

### The models

**Fig 3:**
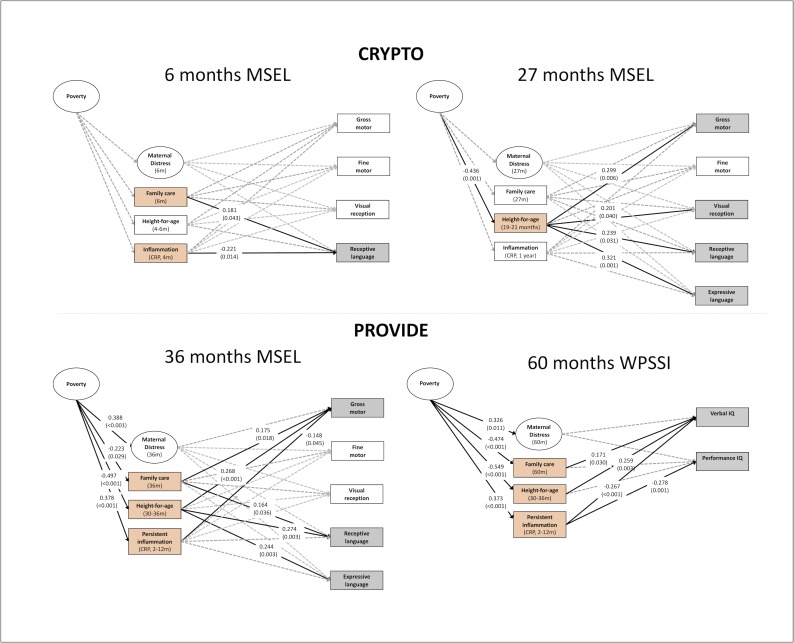
Results from the four path models. Structural Equation Models estimating associations between poverty at birth, hypothesized mediators, and child development. Poverty is defined as a latent factor based on income-to-needs, assets and housing risks. HAZ is used to index child growth, inflammation is measured as elevated C-reactive protein (CRP) in peripheral blood, maternal distress is measured a latent factor based on self-reported depressive symptoms and stress, and family care is measured as parental engagement in stimulating activities with the child. Significant paths (*p*<0.05) are highlighted with a solid line. We only provide the estimates and *p*-values for significant effects.

Bivariate correlations between the study variables are shown in Table 2. We report on the models without direct effects of poverty (Fig 3).

**Table 2:** Bivariate correlations among study variables in CRYPTO and PROVIDE.

	Poverty	Maternal distress	Family care	HAZ	CRP	IL1-b 18w	IL4 18w	IL6 18w	TNF-a 18w
**CRYPTO**									
**Maternal distress**	0.056								
**Family care**	-0.097	-0.005							
**HAZ (average)**	-0.068	-0.004	0.007						
**CRP 18w **	-0.136	-0.008	0.013	0.009					
**IL1-b 18w **	0.030	0.002	-0.003	-0.002	-0.035				
**IL4 18w **	-0.106	-0.006	0.010	0.007	0.054	-0.072			
**6 months cognitive outcomes**									
**Gross motor**	0.060	-0.158	0.017	0.098	0.073	-0.027	-0.046	*NA*	*NA*
**Visual Reception**	0.195*	-0.053	-0.153°	0.112	-0.002	-0.001	-0.071	*NA*	*NA*
**Fine motor**	0.197*	0.092	-0.057	0.053	-0.033	0.030	-0.168°	*NA*	*NA*
**Receptive language **	0.073	0.037	0.161°	0.081	-0.196*	0.077	-0.092	*NA*	*NA*
**28 months cognitive outcomes**									
**Gross motor**	-0.226**	0.137	0.069	0.134	-0.032	-0.170°	-0.079	*NA*	*NA*
**Visual Reception**	-0.230**	0.120	0.051	0.063	0.099	-0.175*	-0.172°	*NA*	*NA*
**Fine motor**	-0.225*	0.076	-0.026	0.146	0.051	-0.115	-0.136	*NA*	*NA*
**Receptive language **	-0.264**	0.002	0.002	0.202	0.141	-0.136	-0.046	*NA*	*NA*
**PROVIDE**									
**Maternal distress**	0.991**								
**Family care**	-0.174	-0.172							
**HAZ (average)**	-0.447**	-0.442**	0.078						
**Persistently elevated CRP **	0.317**	0.290**	-0.055	-0.142					
**IL1-b 18w**	0.160°	0.045	-0.028	-0.071	0.201*				
**IL4 18 w**	0.126	0.006	-0.022	-0.056	0.198*	0.771**			
**IL6 18w**	-0.069	-0.168°	0.012	0.031	0.109	0.612**	0.648**		
**TNF-a 18w**	0.024	-0.097	-0.004	-0.011	0.167°	0.762**	0.802**	0.661	
**36 months cognitive outcomes**									
**Gross Motor**	0.078	0.019	0.216*	0.196*	-0.188*	0.036	0.097	0.103	0.020
**Visual reception**	-0.162°	-0.126	0.208*	0.102	-0.139	0.056	0.061	0.167	0.034
**Fine motor**	-0.042	-0.005	0.186*	-0.011	-0.077	0.035	-0.012	0.103	0.026
**Receptive language**	-0.258**	-0.102	0.187*	0.184*	-0.066	-0.033	-0.002	0.060	-0.029
**Expressive language**	-0.235**	-0.203*	-0.036	0.226**	-0.210*	-0.148°	-0.101	0.030	-0.107
**60 months cognitive outcome **									
**Performance IQ**	-0.208*	-0.045	0.096	0.091	-0.288**	-0.074	-0.035	0.024	0.016
**Verbal IQ**	-0.467**	-0.339**	0.186*	0.272**	-0.314**	-0.050	-0.088	0.014	-0.031

M = months; HAZ = height-for-age; CRP = C-reactive protein.

** Indicates p<0.01

* indicates p<0.05

°Indicates p<0.1.

#### The model in the 6 months old infants (CRYPTO)

The hypothesized model with paths from poverty to the hypothesized mediators (HAZ, inflammation, maternal distress, and family care), and from HAZ, inflammation, maternal distress, and family care to child outcomes is shown in Fig 3. We exclude the expressive language scale in the analyses predicting 6-month outcomes because of a very narrow distribution in children’s performance with most children scoring within a two-point margin.

The model demonstrated acceptable model fit (χ^2^(38) = 42.268, P = 0.292; CFI = 0.969; RMSEA = 0.029; SRMR = 0.055). We did not find any associations between poverty and child exposure to the hypothesized mediators at 6 months. We did find, however, that inflammation at 4 months was associated with lower receptive language scores at 6 months and that children whose parents reported engaging in more types of stimulating activities had higher receptive language scores at 6 months, compared with children whose parents engaged in fewer activities. Neither HAZ nor maternal distress explained variance in children’s developmental outcomes and no risk exposure explained variation in children’s gross motor, fine motor, visual reception, or receptive language scores at 6 months.

#### The model in the 27 months old infants (CRYPTO)

The hypothesized model with the developmental outcomes from the CRYPTO children’s 27 months visit showed acceptable model fit (χ^2^(46) = 46.103, P = 0.926; CFI = 1.000; RMSEA = 0.004; SRMR = 0.048). In this model we did observe a significant negative relationship between poverty at enrolment and children’s HAZ at 19–21 months, yet we did not see any significant associations between poverty and inflammation at 1 year, maternal distress at 27 months, or family care at 27 months. We also found that higher HAZ scores, in turn, were associated with higher scores on the gross motor, visual reception, receptive language, and expressive language scales. Maternal distress, family care, and inflammation did not show any relationships with a child’s scores on the developmental assessments. Given the association between poverty and HAZ, and between HAZ and child outcomes, we examined four indirect pathways through which poverty may affect gross motor, visual reception, receptive language, and expressive language via children’s HAZ. Two significant indirect effects emerged, namely from poverty to gross motor and expressive language via HAZ (Table 3).

**Table 3:** Indirect effects of poverty on child development via HAZ, inflammation and family care. Bold indicates a significant effect determined by *p*<0.05 and a 95% confidence interval that does not include 0.

	Estimate	SE	95% CI (bootstrapped)		*p*-value
			LL	UL	
**CRYPTO—27 months outcomes**					
**Poverty → HAZ → Gross motor**	**-0.139**	**0.059**	**-0.313**	**-0.039**	**0.019**
Poverty **→** HAZ **→** Visual reception	-0.093	0.055	-0.244	-0.008	0.087
Poverty **→** HAZ **→** Receptive language	-0.111	0.062	-0.286	-0.013	0.073
**Poverty → HAZ → Expressive language**	**-0.149**	**0.062**	**-0.324**	**-0.042**	**0.016**
**PROVIDE—36 months outcomes**					
**Poverty → HAZ → Gross motor**	**-0.145**	**0.047**	**-0.255**	**-0.058**	**0.002**
**Poverty → HAZ → Receptive language**	**-0.154**	**0.058**	**-0.294**	**-0.052**	**0.009**
**Poverty → HAZ → Expressive language**	**-0.133**	**0.052**	**-0.255**	**-0.039**	**0.009**
Poverty **→** Family care **→** Gross motor	-0.042	0.024	-0.111	-0.004	0.082
Poverty **→** Family care **→** Receptive language	-0.040	0.024	-0.117	-0.002	0.134
Poverty **→** Inflammation **→** Gross motor	-0.054	0.032	-0.164	-0.001	0.094
**PROVIDE– 60 months outcomes**					
**Poverty → HAZ → Verbal IQ**	**-0.139**	**0.054**	**-0.267**	**-0.048**	**0.010**
Poverty **→** HAZ **→** Performance IQ	-0.066	0.052	-0.184	0.033	0.210
**Poverty → Family care → Verbal IQ**	**-0.080**	**0.040**	**-0.177**	**-0.011**	**0.044**
**Poverty → Inflammation → Verbal IQ**	**-0.100**	**0.040**	**-0.202**	**-0.035**	**0.012**
**Poverty → Inflammation → Performance IQ**	**-0.105**	**0.043**	**-0.209**	**-0.034**	**0.015**

#### The model in the 36 months olds (PROVIDE)

The model predicting 36-month outcomes in the PROVIDE children showed acceptable model fit (χ^2^(46) = 35.029, P = 0.995; CFI = 1.00; RMSEA = 0.000; SRMR = 0.043). In the 36-month olds, we found that greater poverty was associated with lower child HAZ, and more frequent inflammation, higher levels of maternal distress, parental engagement in fewer stimulating activities with the child, as hypothesized.

HAZ was the most consistent predictor of child outcomes and was positively associated with gross motor, receptive language, and expressive language scores. Family care was positively associated with gross motor and receptive language scores. Persistent inflammation (more frequent elevations in CRP across 5 blood draws collected over the first two years of life) was associated with lower gross motor scores. There was no significant relationships between maternal distress and child developmental outcomes at 36 months. With regard to the indirect effects of poverty, we found that poverty was indirectly associated with variation in gross motor, receptive language, and expressive language via the effect of poverty on HAZ. The indirect effects of poverty on gross motor and receptive language via family care and inflammation did not reach significance (see Table 3).

#### The model in the 60 months olds (PROVIDE)

The model predicting 60-month outcomes from the second visit with the PROVIDE children showed acceptable model fit (χ^2^(35) = 32.327, P<0.598; CFI = 1.00; RMSEA = 0.000; SRMR = 0.052). Similar to the findings at 36 months, we found that poverty at enrolment was associated with lower HAZ at 30–36 months, more persistent inflammation over the first two years of life, more maternal distress at 60 months, and less family care at 60 months. With regard to child outcomes, we found that more persistent inflammation was associated with lower verbal and performance IQ on the WPPSI. Higher HAZ and family care scores were associated with higher verbal IQ. The indirect effects analyses showed that poverty explained variation in verbal IQ via its effect on family care, HAZ, and inflammation. Poverty also explained variation in performance IQ via childhood inflammation (see Table 3).

## Discussion

In this study, we demonstrated that the development of children growing up in poverty in Bangladesh is impacted not only by the financial and material constraints typically associated with poverty, but by a wide array of risk exposures that impact children’s social environment, child growth (HAZ), and inflammation (elevated CRP). Developmental outcomes were assessed at four ages: at 6 months and 27 months in the CRYPTO cohort, and at 36 months and 60 months the PROVIDE cohort. In CRYPTO we found that inflammation and family care explained variation in children’s receptive language at 6 months. At 27 months we found significant associations between HAZ and gross motor, visual reception, and language (receptive and expressive) scores. We did not see any associations between inflammation, family care or maternal distress and child outcomes at 27 months. In PROVIDE we found significant associations between HAZ, inflammation, family care and child outcomes related to gross motor and language (receptive and expressive) at 36 months. At 60 month we found associations between HAZ, inflammation and family care and children’s performance and verbal IQ. Only one outcome, language, was significantly associated with one or more risk exposures across all of the four ages. This may suggest that language is particularly susceptible to poverty-related risks exposures, and that the susceptibility or sensitivity of language development starts before 6 months of age.

A key aim of the study was to examine whether children’s HAZ, a measure of child’s growth status that we use as a putative index of a child’s early nutritional environment [3], inflammation measured as elevated CRP at one or multiple time points, family care measured as caregiver engagement in stimulating activities with the child, and maternal distress measured as maternal symptoms of depression and stress accounted for some of the effects of poverty on children’s developmental outcomes. We found evidence of indirect effects of poverty via HAZ, inflammation and family care on children’s developmental outcomes including motor functions, visual reception, and language at 36 and 60 months. Although previous studies have highlighted the importance of caregiving as a mediator of the effect of poverty on children’s development [3, 18], few studies shown independent associations between co-occurring psychosocial and biological risks and child outcomes in settings of poverty.

A child’s HAZ was found to be the main predictor of child outcomes at 27 and 36 months, showing association with gross motor, expressive language, and receptive language at both timepoints, and with visual reception at 27 months. Furthermore, children’s HAZ at 30–36 months predicted verbal IQ at 60 months. We also found evidence of indirect effects of poverty on child development via HAZ at 27, 36, and 60 months suggesting that child growth is an important mediator of poor child outcomes among child growing up in poverty. The possible biological mechanisms linking poor growth to poor developmental outcome are multiple but likely involve effects of micronutrient and macronutrient deficiencies on neural development and functioning [2].

The finding of associations between inflammation and children’s developmental outcomes at 6, 36, and 60 months extends previous findings of relationships between childhood inflammation and developmental outcomes in children aged 18 to 24 months [5, 6] by showing that inflammation may impact a child’s language development as early as 6 months. The absence of an effect of inflammation on developmental outcomes at 27 months may reflect the time gap of one year between the assessment of CRP and the developmental outcomes. This is a longer gap compared with the 2 month time-gap between CRP at 4 months and language outcomes at 6 months. In CRYPTO we used a single CRP assessment in CRYPTO whereas we used repeated assessments to measure persistent inflammation in PROVIDE. Persistent, as opposed to acute inflammation may have more pervasive impacts on neural and cognitive development which may explain the more persistent finding of associations between inflammation and child outcomes in PROVIDE [25]. Although more work using neuroimaging and animal models is needed to elucidate the actual biological mechanism linking inflammation to cognitive outcomes, a key pathway is likely to involve effects of peripheral inflammation on neural development and functioning [26]. For example, peripheral mediators of inflammation such as inflammatory cytokines can access the brain and activate an inflammatory response within the central nervous system which in turn may have long-term effects on children’s neural and behavioral development [26]. Inflammation can also impact neural growth factors which in turn may impact neural and cognitive development [25].

The finding that family care predicted developmental outcomes as early as 6 months adds a wealth of evidence highlighting the importance of the social environment on children’s cognitive development, including previous evidence from Bangladesh where family care was found to explain developmental outcomes at 18 months [18].

We did not observe any effects of maternal distress on children’s developmental outcomes. This is in contrast to previous studies that have shown negative effects of maternal depression on child development in infants as young as 3 months [13]. Other studies conducted in global low resource settings also did not find effects of maternal depressive symptoms on the MSEL in 12–54 months old infants in Benin [27] and Uganda [28]. It may be that the impact of other risk exposures that are common in global low resource environments, such as malnutrition and infectious disease, may mask potential effects of poor maternal mental health. Alternatively, it may be that developmental outcomes captured by the MSEL lack the sensitivity to capture the effects of maternal distress compared with other developmental assessment tools, or vice versa—that our measure of maternal distress lacks the sensitivity to capture variability on the MSEL. Although a lot of care was taken when translating the questionnaires and when interviewing mothers, cultural differences in perceptions and conceptualizations of stress and depression, or challenges in obtaining accurate self-reports, may affect our ability to detect developmental impacts on the children. More objective measures of maternal stress and depression including cortisol or inflammatory markers would be of great values in future studies.

The relative scarcity of associations between risk exposures and developmental outcomes at 6 months relative to later in development is noticeable and may be explained by a number of factors. Firstly, we note that the 6 months old infants on average scored in the normal to above-normal range on the MSEL. At 6 months the infants have a mean score of 105 and the majority of scores fell above or within what would be considered the normal range according to the US standardization. However, from 27 months onwards we observed a steep drop in children’s performance to what would be considered below normal range in a US population (Fig 2). Interestingly, this pattern is in line with observations from longitudinal studies from Japan and the United States. The longitudinal study from Japan examined growth trajectories in MSEL scores collected between ages 1 and 24 months and found that the divergence in T-scores on the MSEL subscales widened over development [29]. The longitudinal study from the United States followed native American children who shared socioeconomic characteristics with our cohorts (namely low income and low parental education), and showed a similar pattern whereby children’s MSEL scores were around the US average at 6 months but dropped between age 6 and 15 months [30]. We suggest that the increasing divergence on the MSEL with children’s age may reflect one of three scenarios: 1) an increasing ability of the MSEL to differentiate between developmental trajectories as children age due to increasing measurement accuracy, 2) a developmental effect whereby children fall behind on developmental outcome gradually over time due to the acceleration of developmental milestone achieved as children age, 3) Developmentally dependent and/or cumulative effects of risk and protective factors. Children may, for instance, need a certain age or a certain amount of risk exposure before development is noticeably impacted by e.g., maternal distress. With regard to protective factors that may attenuate effects of poverty on early child development. we note that most of the infants in our cohort were breastfed until they were at least 6 months old. Breastfeeding is a known protective factor against both malnutrition and infection and may explain the absence of relationships between poverty and HAZ and inflammation at 4–6 months in CRYPTO.

Despite the typical performance of the 6 months old infants and the scarcity of associations between poverty-related factors and children’s developmental outcomes, we emphasize that absence of exposure-behavior associations at 6 months does not necessarily suggest that early risk exposures do not affect children at this age. It may well be that effects are simply too subtle to be measured early in life and thus remain “latent” until the child reaches (or fails to reach) certain developmental milestones. Indeed, many behavioral tools may lack sensitivity to detect developmental differences in young children due to the limited behavioral repertoire, and hence the limited number of abilities that can be tested at young ages. Neuroimaging methods, such as electroencephalography (EEG) and functional near-infrared spectroscopy (fNIRS), may have increased sensitivity to detect variability in early developmental outcomes. Studies of variation in spectral power in baseline/ task-free EEG have, for instance, found variation in EEG power in certain frequency bands in children of low socioeconomic status (SES) compared with children with higher SES identifiable as early as one year of age [31].

A number of limitations to the current study should be acknowledged. First, due to the study design, we were unable to adequately correct for potential effects of pre- and perinatal events that may relate to both poverty and developmental outcomes. Second, the sample size is relatively limited for SEM. We were therefore unable to explore potential interactions among risk exposures to examine, for instance, moderation and cumulative effects of multiple risk exposures although such interaction are likely to be important contribute to the mechanistic pathways through which poverty affects child development. Third, we were unable to follow the same children across all four time points and depended on cross sectional results from two cohorts. Forth, the use of cognitive assessments (MSEL and WPPSI), both of which were developed for English speakers in the United States, may have implications for external validity. Fifth, the collection of CRP assays differed between the two cohorts. While we hypothesize that repeated inflammation likely impacts child development more than a single acute episode [25] we were only able to create a score of the cumulative burden of inflammation and thus used a single time-point measure of inflammation in CRYPTO. This affects our ability to draw general conclusions across the two cohorts and may have affected our ability to detect effects of inflammation on developmental outcomes at 27 months in CRYTPO. That said, we note that there is no defined cut off for *when* inflammation has the potential to impact cognitive development. The split between children in the 50^th^ highest and lowest percentile for their CRP concentration in the PROVIDE cohort was based on a previous published approach to categorizing CRP but it does not allow us to explore a sensitive cut off for when inflammation has adverse effects on child development.

Still, the current study adds to the child development literature by shedding light on important mediating factors that contribute to poor developmental outcomes among children growing up in extreme poverty. We also begin to define the time frame within which the adverse effects of poverty and related risks may emerge, although longitudinal studies are needed to track developmental changes within the same children over a longer period of time.

## Conclusion

The current study identified key risk exposures that explain variation in developmental outcomes among children living in urban poverty in Bangladesh. We found that inflammation at 4 months was associated with lower receptive language scores at 6 months whereas more family care was associated with higher receptive language scores at 6 months. This seems to suggest that the impact of risk exposures on language start early in life. Among the older children we found that HAZ (a putative, but widely used index of children’s early nutritional environment [3]), inflammation (repeatedly elevated CRP over the first year of life), and the family caregiving environment each independently contributed to variation in children’s developmental outcomes 36 and 60 months. This study thus provides empirical support for the importance of holistic and integrated interventions that target multiple aspects of children’s biological and psychosocial environments including nutrition, hygiene, and stimulating caregiver-child interactions.

## Supporting information

S1 Text Cultural adaptation of Mullen Scales of Early Learning (MSEL). Overview of changes made to the adapt the MSEL to the local context.
(DOC)

S1 Table Factor loadings from confirmatory factor analyses. (DOC)
