# Supplementary material for: Child development in the context of biological and psychosocial hazards among poor families in Bangladesh
Source: PLoS One. 2019 May 6;14(5):e0215304. doi: 10.1371/journal.pone.0215304 (PMC6502452; doi:10.1371/journal.pone.0215304)
Supplement: S1 Text — Overview of changes made to the adapt the MSEL to the local context. (DOC) [file pone.0215304.s001.doc]

**S1 Text**

**SUPPLEMENTAL MATERIAL FOR:**

Child development in the context of biological and psychosocial hazards among poor families in Bangladesh

Sarah K. G. Jensen^1,2, ,#a^, Fahmida Tofail^3^, Rashidul Haque^3^, William A. Petri, Jr.^4^, and Charles A. Nelson, III^1,2,5^

^1^ Boston Children’s Hospital, Boston, Massachusetts, United States of America

^2^ Harvard Medical School, Boston, Massachusetts, United States of America

^3^ ICDDR,B, Dhaka, Bangladesh

^4^ University of Virginia, Infectious Diseases & International Health, Charlottesville, Virginia, United States of America

^5^ Harvard Graduate School of Education, Cambridge, Massachusetts, United States of America

**^#^**^a^Current address: School of Social Work, Boston College, Chestnut Hill, Boston, United States of America

Corresponding Author:

[sarahkgeorg@gmail.com](mailto:sarahkgeorg@gmail.com) (SKGJ)

**S1 Appendix: Cultural adaptation of Mullen Scales of Early Learning (MSEL)**

**Receptive Language**

- Item: RL-30: Q-9

Note; There are only 3 types of coins in Bangladeshi culture.

**Change:** We used those 3 coins for testing children instead of 4 coins as instructed in this item.

**Expressive Language**

**Picture changes**

- Item: EL-18: Some pictures are not familiar in Bangladeshi culture. We used the following pictures provided in the stimulus book for the same item.
  - - Phone:
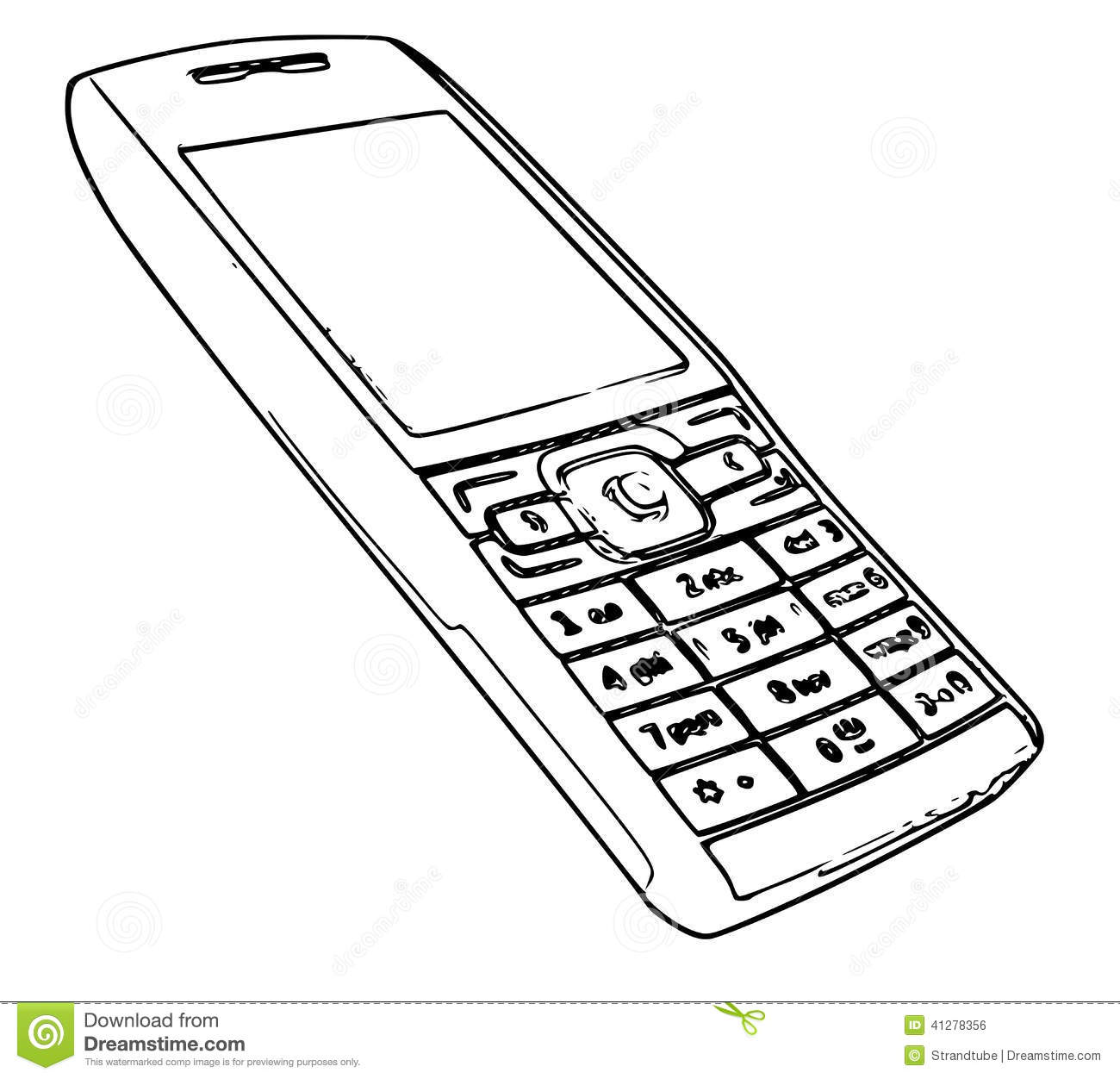
 (Changed picture)
    - Ball:
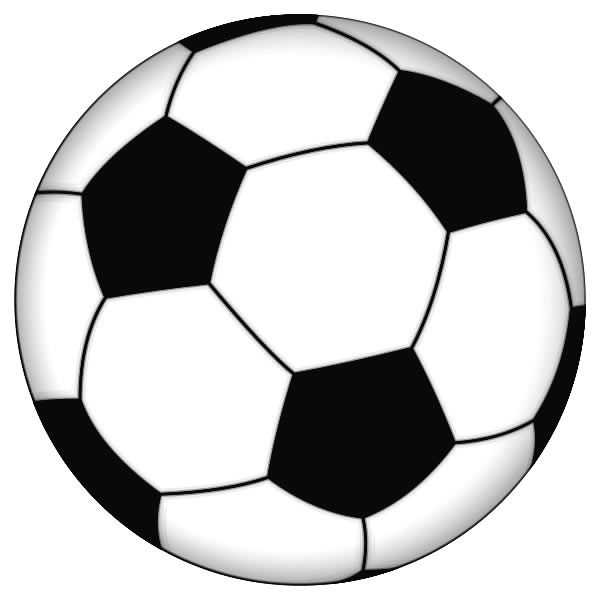
 (Changed picture)
    - Bag:
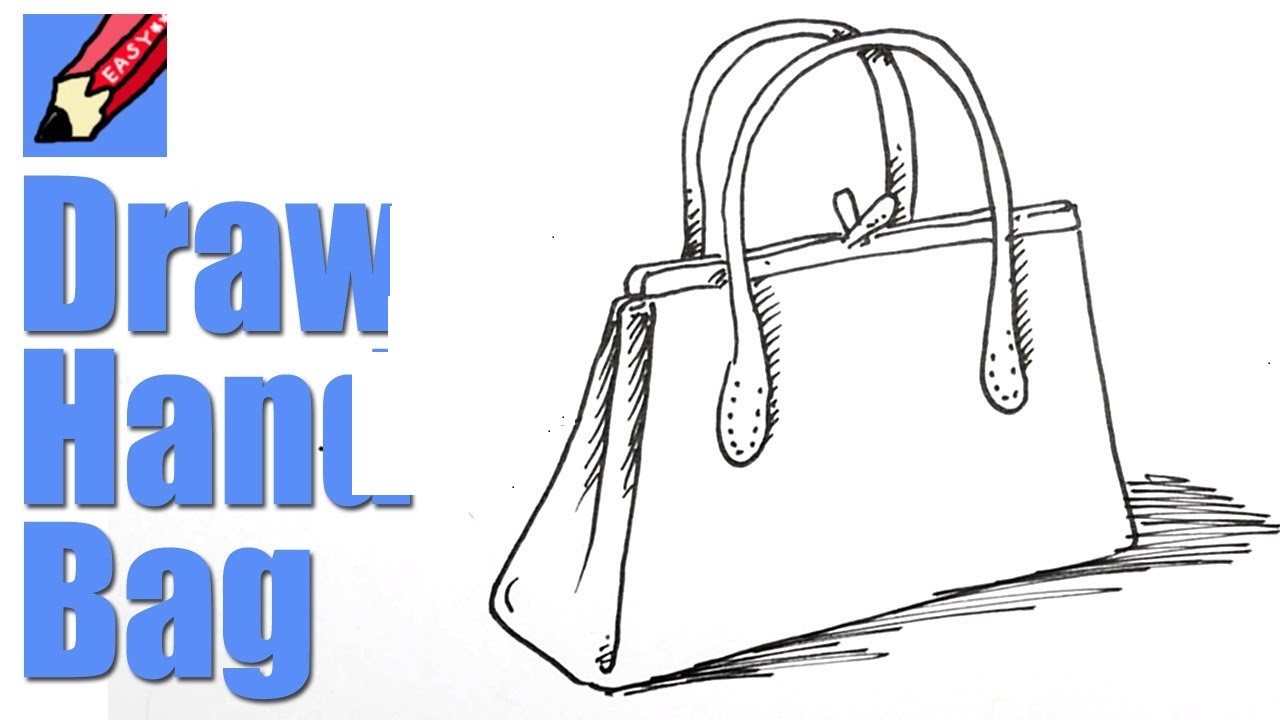
 or
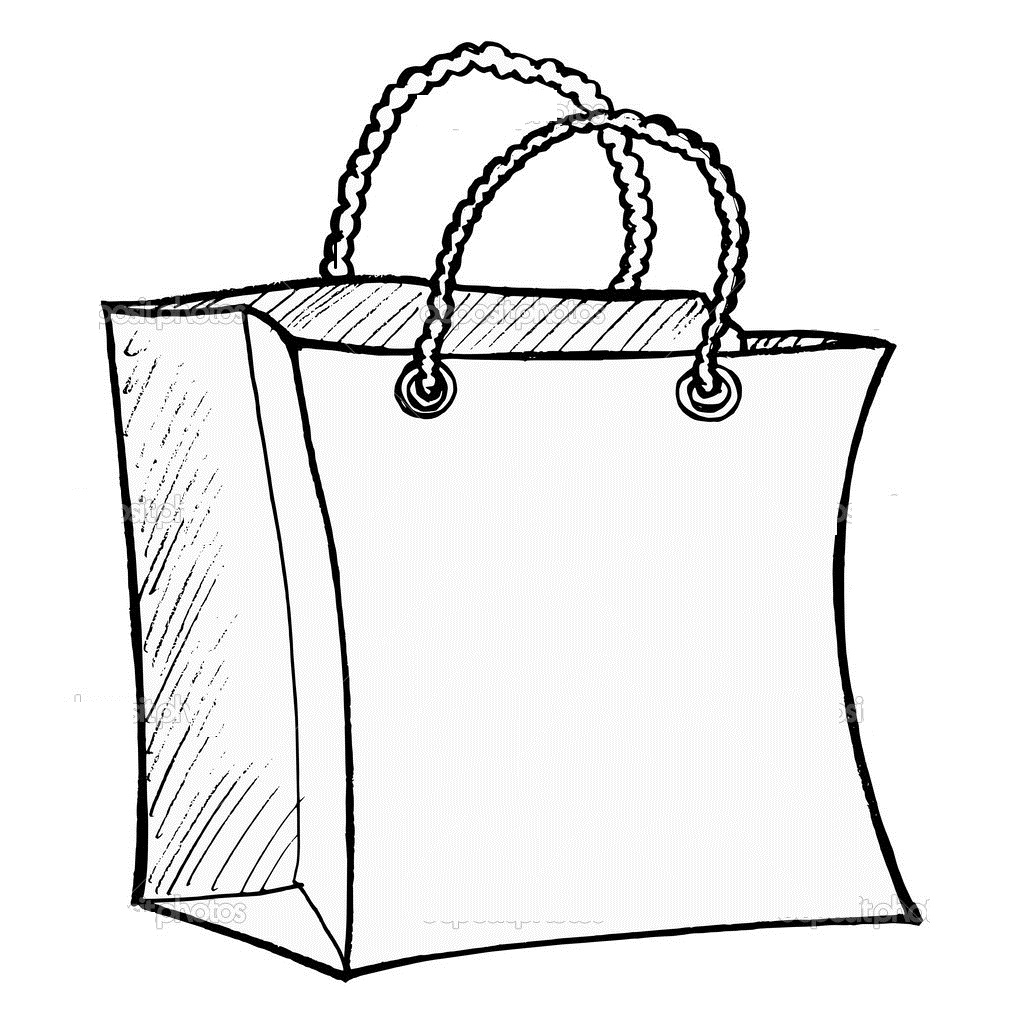
 (Changed picture)
    - Chimney: (Mosque)
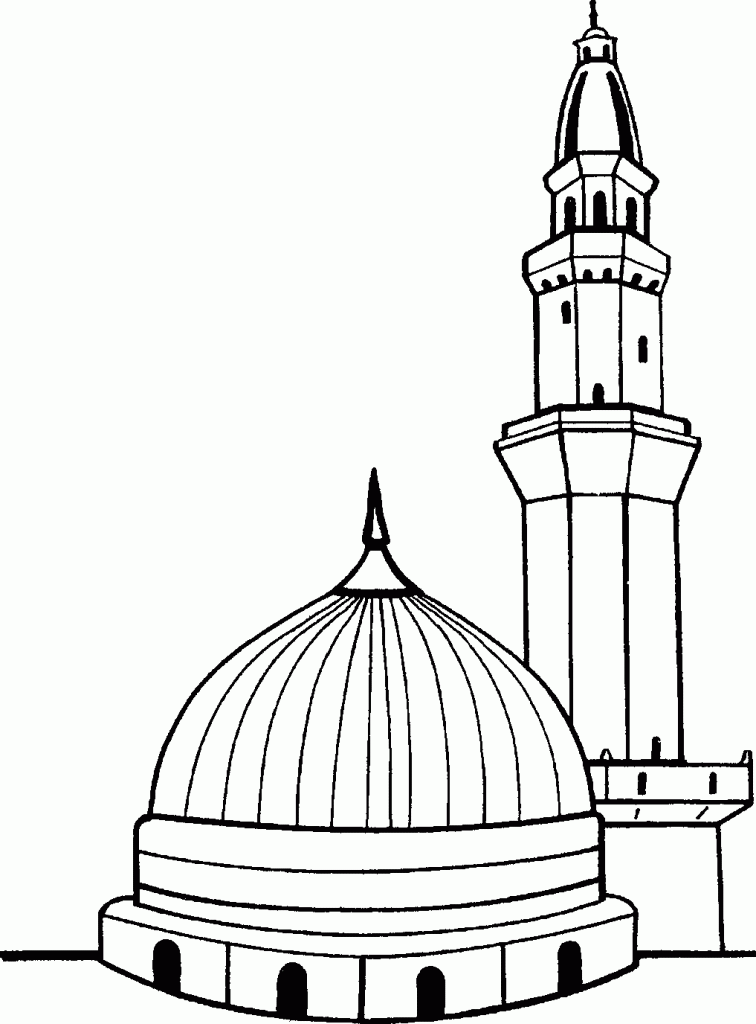
 (Changed picture)
    - Lamp:
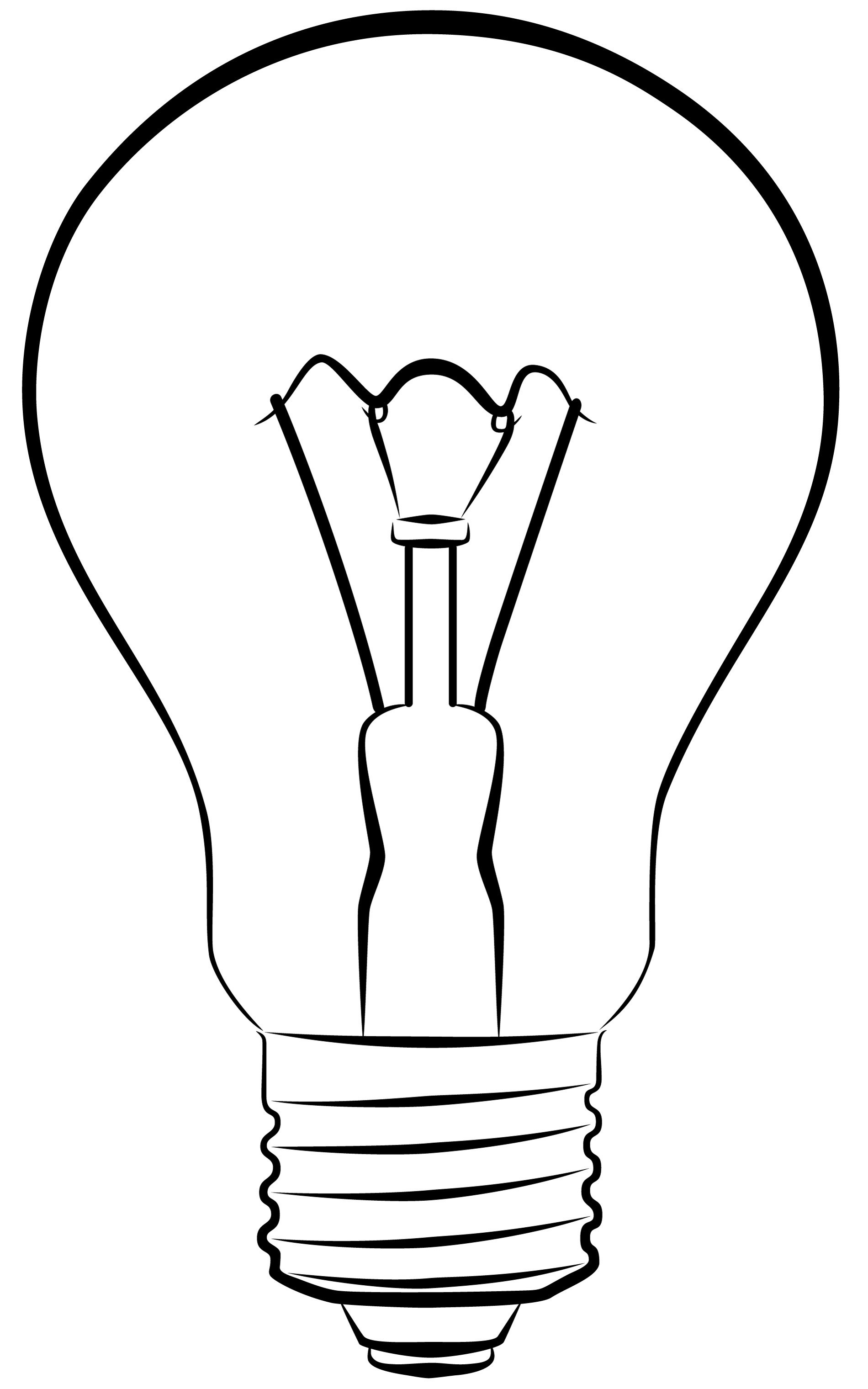
 (Changed picture)
- Item: EL-23: Q-1--- Children in Bangladeshi culture are not familiar with the word “Thirsty”.

**Change:** we have to change the question to “*What do you have when your throat dries up (gola shukhaye gele/ testa paile)*? “ The any kind of drink is a correct answer

- Item: EL-24:

Q-5--- Children in Bangladeshi culture are not familiar with the word “Dinner/ Supper”.

**Change:** “food for night-*Rater Khaowa*”

Q-10--- Children in Bangladeshi culture are not familiar with the word “Tent”.

Change: Replaced- “A house made of wood: a *Nest* made of _____”

- Item: EL-27: Q-8--- Children in Bangladeshi culture are not familiar with the word “Lifeguard”.

Change: “Why shouldn’t you swim at the beach if there’s *no adult accompanying*?”

- Item: EL-28:

Q-2--- Children in Bangladeshi culture are not familiar with the word “Mailman”.

Change: “My dog barks everyday when *someone knocks the door*”

“Dorjaye keu toka dile amar kukur protidin gheu gheu kore dake”

Q-3--- Children in our culture are not familiar with the concept of “Sliding down the hill”

Change “*During play we slide down from slope in our courtyard*”

“khelar shomoye amra uthane uchu dhal theke pisla kahye niche nami”
