# Supplementary material for: Child development in the context of biological and psychosocial hazards among poor families in Bangladesh
Source: PLoS One. 2019 May 6;14(5):e0215304. doi: 10.1371/journal.pone.0215304 (PMC6502452; doi:10.1371/journal.pone.0215304)
Supplement: S1 Table — (DOC) [file pone.0215304.s002.doc]

**S2 Table**

**SUPPLEMENTAL MATERIAL FOR:**

Child development in the context of biological and psychosocial hazards among poor families in Bangladesh

Sarah K. G. Jensen^1,2, ,#a^, Fahmida Tofail^3^, Rashidul Haque^3^, William A. Petri, Jr.^4^, and Charles A. Nelson, III^1,2,5^

^1^ Boston Children’s Hospital, Boston, Massachusetts, United States of America

^2^ Harvard Medical School, Boston, Massachusetts, United States of America

^3^ ICDDR,B, Dhaka, Bangladesh

^4^ University of Virginia, Infectious Diseases & International Health, Charlottesville, Virginia, United States of America

^5^ Harvard Graduate School of Education, Cambridge, Massachusetts, United States of America

**^#^**^a^Current address: School of Social Work, Boston College, Chestnut Hill, Boston, United States of America

Corresponding Author:

[sarahkgeorg@gmail.com](mailto:sarahkgeorg@gmail.com) (SKGJ)

**S1 Table. Factor loadings from confirmatory factor analyses**

Analyses conducted in Mplus version 7.

| **Poverty at enrollment** | **Estimate (indicator 1)** | **Estimate (indicator 2)** | **Estimate (indicator 3)** |
| --- | --- | --- | --- |
|  | **Income-to-needs quatiles** | **Cumulative household assets** | **Housing risk index** |
| CRYPTO (n=130) | 0.623 (p<0.001) | 0.337 (p=0.002) | 0.638 (p<0.001) |
| PROVIDE (n=130) | 0.559 (p<0.001) | 0.672 (p<0.001) | 0.712 (p<0.001) |
| **Maternal distress** |  |  |  |
|  | **Depressive symptoms** | **Percieved stress** |  |
| CRYPTO 6 months (n=129) | 0.711 (p<0.001) | 0.718 (p<0.001) | - |
| CRYPTO 27 months (n=116) | 0.825 (p<0.001) | 0.754 (p<0.001) | - |
| PROVIDE 36 months (n=130) | 0.718 (p=0.014) | 0.677 (p=0.064) | - |
| PROVIDE 60 months (n=116) | 0.901 (p<0.001) | 0.785 (p<0.001) | - |
